# Comparison of the Ahmed glaucoma valve with the Baerveldt glaucoma implant: a meta-analysis

**DOI:** 10.1186/s12886-015-0115-y

**Published:** 2015-10-13

**Authors:** Yi-Wen Wang, Ping-Bao Wang, Chao Zeng, Xiao-Bo Xia

**Affiliations:** Department of Ophthalmology, Xiangya Hospital, Central South University, #87 Xiangya Road, Changsha, Hunan 410008 China; Department of Orthopaedics, Xiangya Hospital, Central South University, Changsha, Hunan Province China

**Keywords:** Glaucoma, Ahmed glaucoma valve implantation, Baerveldt glaucoma implant, Meta-analysis

## Abstract

**Background:**

This study aims to compare the efficacy and safety of the Ahmed glaucoma valve (AGV) with the Baerveldt glaucoma implant (BGI) in glaucoma patients.

**Methods:**

Databases were searched to identify studies that met pre-stated inclusion criteria, involving randomized controlled clinical trials (RCTs) and non-randomized controlled clinical trials. Treatment effect was analyzed using a random-effect model.

**Results:**

Ten controlled clinical trials (1048 eyes) were analyzed, involving two RCTs and eight retrospective comparative studies. Short-term results (6–18 months) and long-term results (>18 months) were analyzed separately. There was no significant difference in the success rate for short-term follow-up between the AGV and BGI groups (5studies, 714 eyes, odds ratio [OR]: 0.97; 95 % confidence interval [CI]: 0.56, 1.66; *P* = 0.90). For long-term pooled results (7studies, 835 eyes), the success rate of AGVs was lower than that of BGIs (OR: 0.73; 95 % CI: 0.54, 0.99, *P* = 0.04), However, subgroup and sensitivity analyses did not show a significant difference in the success rate between the two groups (*P* ≥0.05). The AGV group had a higher mean intraocular pressure than the BGI group in short-term (6 studies, 685 eyes, weighted mean difference [WMD]: 2.12 mmHg; 95 % CI: 0.72–3.52; *P* <0.05) and long-term pooled results (7 studies, 659 eyes, WMD: 1.85 mmHg; 95 % CI: 0.43, 3.28; *P* = 0.01). The BGI group required fewer glaucoma medications after implantation than the AGV group in two follow-up periods (all *P* <0.05). The AGV was found to be associated with a significantly lower frequency of total complications (8 studies, 971 eyes, OR: 0.67; 95 % CI: 0.50–0.90; *P* = 0.007) and severe complications (8 studies, 971 eyes, OR: 0.57; 95 % CI: 0.36–0.91; *P* = 0.02) than the BGI.

**Conclusions:**

The study showed no significant difference in success rate between the two groups. The BGI was more effective for control of intraocular pressure and required fewer medications than the AGV, but the AGV had lower incidence of total and severe complications than the BGI.

**Electronic supplementary material:**

The online version of this article (doi:10.1186/s12886-015-0115-y) contains supplementary material, which is available to authorized users.

## Background

Glaucoma is the leading cause of irreversible blindness worldwide. Because conventional trabeculectomy and glaucoma medicines result in low success rates [1, 2], glaucoma drainage implants (GDIs) have been used with increasing frequency in the management of refractory glaucoma. In 1969, Molteno [3] invented the first of many glaucoma implants. The Ahmed glaucoma valve (AGV) and Baerveldt glaucoma implant (BGI) are currently two of the most commonly used implants for aqueous drainage. Both of them reduce intraocular pressure (IOP) by draining aqueous humor through a tube to a subconjunctival end plate. The AGV contains a one-way valve, which opens in response to a pressure increase in the anterior chamber, and thus helps to reduce the risk of complications, such as hypotony [4, 5]. The BGI, which has no valves, is available in three models according to different surface areas of the end plate (500 mm^2^, 350 mm^2^, and 250 mm^2^). A review by Patel et al. [6] concluded that the AGV has similar success rates and IOP-lowering effects as the BGI. However, a study by Budenz et al. showed that BGI implants produce greater long-term reduction in IOP [7]. Therefore, in the present study, we aimed to determine the efficacy and safety of these two devices for treating patients with glaucoma.

## Methods

The study was approved by the ethics committee at Xiangya Hospital, Central South University, and was conducted in accordance with the Protocol of Helsinki.

### Search strategy and trial selection

We searched PubMed, EMBASE, and Cochrane Controlled Trials Register databases (up to February, 2015) using the following search terms: “glaucoma,” “ocular hypertension,” “intraocular pressure,” “Ahmed,” and “Baerveldt.” The publication dates and languages were not limited, and we identified references of retrieved articles and reviews (Additional file 1). Screening of the articles was performed independently by two reviewers. Studies meeting the following criteria were considered eligible for our meta-analyses: (1) a study design involving comparative clinical trials, including randomized controlled clinical trials (RCTs) and non-randomized controlled clinical trials (non-RCTs); (2) eyes diagnosed with glaucoma undergoing the AGV or BGI; and (3) at least one of the following reported outcomes: success rate, number of glaucoma medicines, mean IOP, and occurrence of adverse events. Exclusion criteria were as follows: (1) case reports, reviews, animal trials, and letters to the editor; (2) studies involving surgery combined with other glaucoma surgeries; (3) studies that implanted two or more GDIs; and (4) studies involving eyes undergoing GDI replacement surgery.

### Data extraction and qualitative assessment

Article quality and extracted data were assessed by two independent readers. Any disagreements were resolved by discussion. The information collected included the first author, publication year, study design, participants (number, age, and sex), GDI model, follow-up time, and baseline IOP.

Quality assessment of the RCTs was performed using Cochrane Collaboration’s tool to assess risks of bias [8], including selection bias, performance bias, detection bias, attrition bias, reporting bias, and other biases. Every bias item was associated with a level of risk (high, low, or unclear). The quality of non-RCTs was evaluated according to an assessment system for non-randomized studies reported by the Chinese Cochrane Centre [9]. The checklist of the system consisted of six items: methods of grouping, methods of blinding, inclusion of all patients, baselines, standards of diagnosis, and control of confounding factors. Because bias of selective reporting was not included in this system, we added the item in assessment. Each item was worth 0–2 points, with a maximum total of 14 points. The overall quality of evidence was evaluated using the GRADE system (performed by GRADEpro3.6, http://cebgrade.mcmaster.ca/Introduction/index.html) [10].

### Statistical analysis

Data analysis was performed using Review Manager 5 software (RevMan 5, The Cochrane Collaboration, Oxford, UK). For dichotomous outcomes, odds ratios (ORs) were calculated. For continuous outcomes, the mean and SD were used to calculate weighted mean differences (WMDs). The heterogeneity of effect size was evaluated by the chi-square test. I^2^ statistics and *P* value were calculated. *P* >0.1 was considered as no significant heterogeneity. Results were pooled using the random-effect model in a meta-analysis. To evaluate publication bias, we performed Begg’s test [11] and inspected funnel plots. *P* <0.05 was considered statistically significant. A sensitivity analysis was conducted to confirm the stability of the meta-analysis results. PRISMA checklist for this meta-analysis can be obtained in Additional file 2.

## Results

**Fig. 1:**
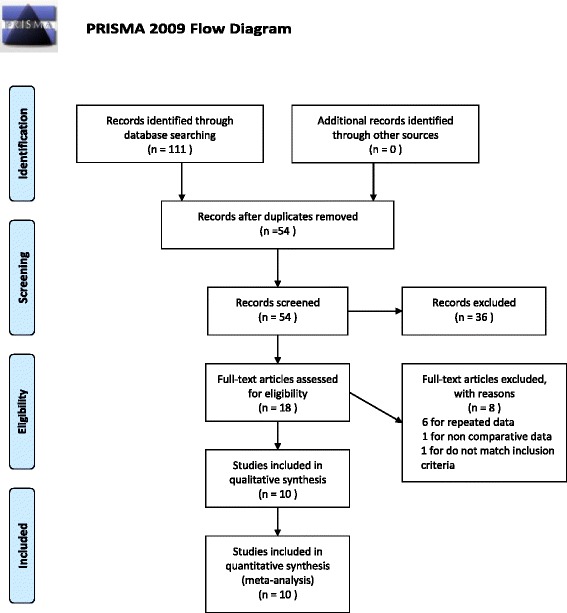
Flow diagram for the selection of included trials

The study identification process is illustrated in Fig. 1. A total of 54 articles were identified by search strategies after duplicates were removed. No study reporting other outcomes was found in comparing the two interventions. Ten articles that enrolled a total of 1048 eyes (486 in the AGV group and 562 in the BGI group) were included in our meta-analysis [7, 12–20]. Two of them were RCTs and the remaining studies were retrospective comparative studies. Two of the included retrospective comparative studies (Tesser et al. [16] and Chung et al. [17]) concurrently performed lens extraction (phacoemulsification or extracapsular cataract removal) with intraocular lens (IOL) implantation or secondary IOL implantation. Although we did not limit the types of glaucoma, most patients undergoing implantation were diagnosed with refractory glaucoma. The mean ages ranged from 5 months to 80 years. The male to female sex ratio ranged from 0.57 to 1.67 in the AGV group, and 0.6 to 1.88 in the BGI group. The follow-up time ranged from 8 months to 5 years. Study characteristics are listed in Table 1.

**Table 1:** Baseline characteristics of eligible clinical trials

Author (Year)	Design	Inclusion criteria	Number of eyes	Sex (M/F)	Age (year)	Models of AGV	Models of BGI	IOP (mmHg) Standards of Success	Follow-up duration (months)	Baseline IOP (mmHg)
Budenz DL (2015)	RCT	>18y	A:143	A:73/70	A:65.4 ± 12.8	FP7	350	5≤ IOP ≤21 and ≥20 % reduction	60	A:31.2 ± 11.2
			B:133	B:70/63	B:62.2 ± 14.2					B:31.8 ± 12.5
Christakis PG (2013)	RCT	>18y	A:124	A:65/59	A:65 ± 17	FP7	350	5≤ IOP ≤21* and ≥20 % reduction	36	A:31.1 ± 10.5
			B:114	B:41/73	B:67 ± 15					B:31.7 ± 11.1
El Gendy NM (2012)	Retro	<18y	A:11	A:4/7	A:6.7	S2	250	8≤ IOP ≤24	A:32.4	A:39.8 ± 6.2
			B:20	B:12/8	B:5.4				B:45.6	B:33.8 ± 5.7
Goulet RJ (2008)	Retro	All ages	A:59	A:25/34	A:66.3 ± 15.14	S2	250	5< IOP <22 and ≥20 % reduction	A:20.0 ± 26.7	A:35.3 ± 13.4
			B:133	B:64/69	B:64.3 ± 16.9				B:22.9 ± 19.9	B:35.3 ± 12.9
Tsai JC (2006)	Retro	>18y	A:48	A:18/30	A:69.2	S2	250 and 350	6≤ IOP ≤21 and ≥20 % reduction	48	A:38.5
			B:70	B:36/34	B:62.3					B:34.6
Tesser R (2005)	Retro	<18y, concurrent primary or secondary IOL implantation	A:3	ND	7.6	S2	250 and 350	IOP ≤22	21	A:35 ± 4.6
			B:6							B:31.3 ± 0.8
Chung AN (2004)	Retro	>18y,concurrent Phaco and IOL implant	A:16	15/17	58 ± 16	ND	350	6≤ IOP ≤21	13 ± 5	A:26.2 ± 13.4
			B:16							B:29.7 ± 13.4
Syed HM (2004)	Case control	All ages, Baerveldt implantation were matched case by case with Ahmed valve implantation	A:32	A:20/12	A:58 ± 24	Polypropylene	350	5< IOP <22 and ≥30 % reduction	8–16	A:30.69 ± 10.28
			B:32	B:13/19	B:61 ± 23					B:30.09 ± 9.17
Wang JC (2004)	Retro	All ages	A:18	A:10/8	A:60.0 ± 18.2	S2	250	IOP <22	A:22.2 ± 9.2	A:43.7 ± 9.3
			B:24	B:15/8	B:48.1 ± 23.2				B:22.8 ± 8.7	B:40.1 ± 13.8
Beck AD (2003)	Retro	<2y	A:32	ND	7mon ± 5.1	S2 and S3	250 and 350	IOP <23	A:33.0 ± 25.5	A:32.2 ± 7.0
			B:14						B:24.9 ± 12.9	B:33.5 ± 5.6

*RCT*: prospective randomized controlled trial; *Retro*: retrospective comparative controlled trial; *ND*: no details; *IOL*: Intraocular lens; *Phaco*: phacoemulsification; *IOP*: intra-ocular pressure; *AGV (A)*: Ahmed glaucoma valve group; *BGI (B)*: Baerveldt Glaucoma Implant group

*The study reported set several different IOP criteria (14 mmHg, 18 mmHg, and 21 mmHg). We adopted the criterion of 21 mmHg

Qualitative assessment of these studies is summarized in Tables 2 and 3. Chung et al’s study [17] was assessed with a low quality score (score 5). Tesser et al’s study [16] had an inadequate sample size. Both of these studies concurrently performed lens-related surgeries. To eliminate potential heterogeneity, we performed a sensitivity analysis after removal of data from these two articles.

**Table 2:** List of biases in RCTs

	Random sequence generation	Allocation concealment	Blinding of participants and personnel	Blinding of outcome assessment	Incomplete outcome data	Selective reporting	Other bias
Budenz DL (2015)	low risk	low risk	high risk	high risk	low risk	low risk	/
Christakis PG (2013)	low risk	low risk	high risk	high risk	low risk	low risk	/

**Table 3:** Quality assessment of non-RCTs

	Methods of grouping	Methods of blinding	Inclusion of all patients	Baselines	Standards of diagnosis	Control of confounding factors	Selective reporting	Total score
El Gendy NM (2012)	0	0	0	2	2	1	2	7
Goulet RJ (2008)	0	0	2	2	2	2	2	10
Tsai JC (2006)	0	0	2	2	2	1	0	7
Tesser R (2005)	0	0	2	2	2	0	2	8
Chung AN (2004)	0	0	0	1	2	0	2	5
Syed HM (2004)	0	0	1	2	2	1	1	7
Wang JC (2004)	0	0	1	2	2	0	2	7
Beck AD (2003)	0	0	2	1	2	0	1	6

For studies with results available at different time points, we analyzed short-term results and long-term results separately. For analysis of short-term results, we pooled data during the mean follow-up times between 6 months to 18 months. Data at 1-year time points in long-term studies were also included. Data at final follow-ups of studies with mean follow-up times >18 months were analyzed for long-term results. Subgroup analyses were performed based on patients’ age (children and adults subgroups) and the study design (RCT and non-RCT subgroups). The boundary of age between the children subgroup and adult subgroup was 18 years.

### Success rate

**Fig. 2:**
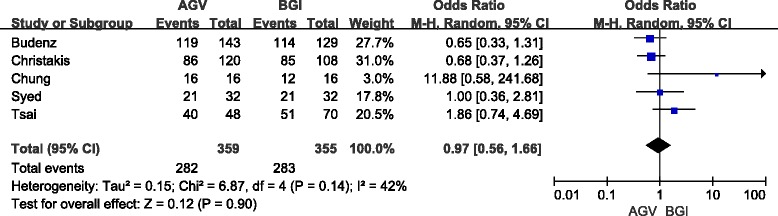
Forest plot of meta-analysis: success rates in short-term follow-up

**Fig. 3:**
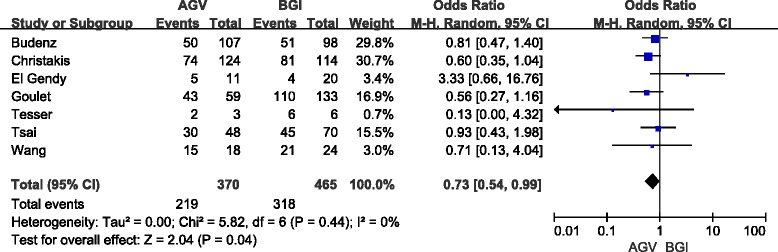
Forest plot of meta-analysis: success rates in long-term follow-up

The definition of success rate was consistent with the original studies with one exception. Christakis et al. [12] reported three sets of results according to different IOP criteria (≤14 mmHg, 18 mmHg, or 21 mmHg). We adopted results using IOP criteria less than 21 mmHg in this article. For the rest of the studies, the crude data was pooled directly based on their original definition of success rate. Five studies (714 eyes) were included in the short-term analyses, and seven studies (835 eyes) were included in the long-term analyses. In short-term follow-up, the success rate in the AGV group was 78.6 % and that in the BGI group was 79.7 %. No significant difference was observed between the two groups (OR: 0.97; 95 % confidence interval [CI]: 0.56, 1.66; *P* = 0.90) (Fig. 2). Sensitivity analyses (4 studies, 682 eyes) yielded a similar result (OR: 0.87; 95 % CI: 0.56, 1.35; *P* = 0.53). In long-term follow-up, the success rate in the AGV group was 59.2 % and that in the BGI group was 68.4 %. Pooled results (OR, 0.73; 95 % CI: 0.54, 0.99) showed a P-value of 0.04, slightly less than the 0.05 threshold (Fig. 3). Therefore, there are some evidence indicate that the success rate for the AGV group was significantly lower than BGI group in long-tern follow-up. Moreover, sensitivity analyses (6 studies, 826 eyes) showed no significant difference between the two groups (OR: 0.74; 95 % CI: 0.55, 1.00; *P* = 0.05). A pooled result from three studies (632 eyes) showed that the number of reoperations for glaucoma in the AGV group was significantly higher than that for the BGI group (OR: 2.70; 95 % CI: 1.54, 4.74; *P* = 0.0005).

A summary of subgroup and sensitivity analyses is shown in Table 4. Although the BGI group showed a higher success rate in total results for long-term follow-ups than the AGV group, subgroup and sensitivity analyses did not show a significant difference between the two groups. The pooled results of the RCT and non-RCT subgroups showed no evidence of statistically significant differences between the two groups for short- and long-term follow-ups. Data from two studies (40 eyes) that focused on children were pooled in long-term follow-up. We found no significant difference in success rate was been observed (OR: 0.96; 95 % CI: 0.04, 21.88, *P* = 0.98) and there was high heterogeneity (I^2^ = 64 %, *P* = 0.1). The large CI suggests that this result may not be reliable. The pooled results of the adult subgroup showed that there was no significant differences in two follow-up times (Table 4). The heterogeneity test showed a lack of significant heterogeneity for total and sensitivity analyses, and RCT, Non-RCT subgroup (I^2^ < 50 %, *P* >0.1).

**Table 4:** Comparison of the success rate

Studies of subgroups	No. of studies	Crude data (n/N)		OR (95 % CI)	Heterogeneity		Test for over effect (*P*)
		AGV	BGI		I^2^ (%)	*P*	
Short-term follow-ups							
Total	5	282/359	283/355	0.97 (0.56–1.66)	42	0.14	0.90
Sensitivity analysis	4	266/343	271/339	0.87 (0.56–1.35)	23	0.27	0.53
RCT	2	205/263	199/237	0.67 (0.42–1.06)	0	0.92	0.09
Non-RCT	3	77/96	84/118	1.63 (0.71–3.74)	24	0.27	0.25
Children	0	/	/	/	/	/	/
Adults	4	261/327	262/323	1.01 (0.50–2.01)	56	0.08	0.99
Long-term follow-ups							
Total	7	219/370	318/465	0.73 (0.54–0.99)	0	0.44	0.04
Sensitivity analysis	6	217/367	312/459	0.74 (0.55–1.00)	0	0.43	0.05
RCT	2	124/231	132/212	0.70 (0.47–1.02)	0	0.46	0.07
Non-RCT	5	95/139	186/253	0.82 (0.45–1.48)	22	0.28	0.51
Children	2	7/14	10/26	0.96 (0.04–21.88)	64	0.1	0.98
Adults	3	154/279	177/282	0.74 (0.52–1.04)	0	0.61	0.08

### IOP

**Fig. 4:**
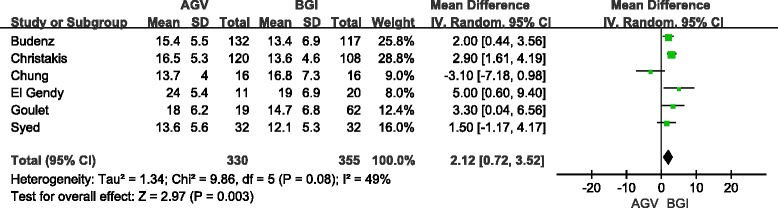
Forest plot of meta-analysis: intraocular pressures in short-term follow-up

**Fig. 5:**
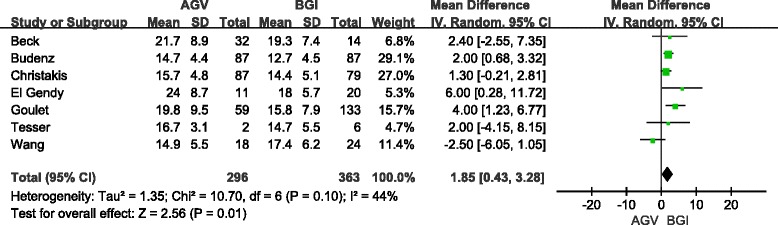
Forest plot of meta-analysis: intraocular pressures in long-term follow-up

We pooled the mean IOPs for the two groups because all articles reported the absolute IOP after the operation. Detail data of total and subgroup analyses are shown in Table 5. In short-term follow-up, the difference in the pooled mean IOP from six studies (685 eyes) for the AGV group compared with the BGI group was 2.12 mmHg (95 % CI: 0.72, 3.52), which was statistically significant (*P* = 0.003, Fig. 4). Significant heterogeneity was observed (I^2^ = 49 %, *P* = 0.08). Sensitivity analyses showed that the overall WMD did not substantially change, and no evidence of significant heterogeneity was observed (I^2^ = 0 %, *P* = 0.6). In long-term follow-up, the difference in the pooled mean IOP from seven studies (659 eyes) for the AGV group compared with the BGI group was 1.85 mmHg (95 % CI: 0.43, 3.28), which was statistically significant (*P* = 0.01, Fig. 5). However, significant heterogeneity was observed (I^2^ = 44 %, *P* = 0.1). The result of Sensitivity analyses (excluded two studies) consisted with the total group (included all eligible studies), but heterogeneity was still significant (I^2^ = 53 %, *P* = 0.06).

**Table 5:** Comparison of postoperative IOP

Studies of subgroups	No. of studies	No. of eyes	WMD (95 % CI)	Heterogeneity		Test for over effect (*P*)
				I^2^ (%)	*P*	
Short-term follow-ups						
Total	6	685	2.12 (0.72–3.52)	49	0.08	0.003
Sensitivity analysis	5	653	2.58 (1.70–3.46)	0	0.60	0.000
RCT	2	477	2.54 (1.54–3.53)	0	0.38	0.000
Non-RCT	4	208	1.68 (−1.27–4.63)	64	0.04	0.26
Children	1	31	5.00 (0.60–9.40)	/	/	0.03
Adults	3	509	1.44 (–0.76–3.65)	74	0.02	0.20
Long-term follow-ups						
Total	7	659	1.85 (0.43–3.28)	44	0.1	0.01
Sensitivity analysis	6	651	1.86 (0.30–3.41)	53	0.06	0.02
RCT	2	340	1.70 (0.70–2.69)	0	0.49	0.001
Non-RCT	5	319	2.18 (−0.91–5.27)	60	0.04	0.17
Children	3	85	3.42 (0.22–6.62)	0	0.56	0.04
Adults	Same as RCT group

The pooled results from the RCT group were similar to the total group in short-and long-term follow-ups, with no statistically significant heterogeneity. No difference in IOP was observed between the BGI and AGV groups in the non-RCT subgroup in short-term follow-up (4 studies, 208 eyes, WMD: 1.68; 95 % CI:-1.27, 4.63; *P* = 0.26) and long-term follow-up (5 studies, 319 eyes, WMD: 2.18, 95 % CI: −0.91, 5.27; *P* = 0.17). Significant heterogeneity was observed in the non-RCT group (*P* <0.1). The results of the children subgroup analysis consisted with the total group. For short-term follow-up, adult subgroup analysis included two RCTs and one non-RCT study (total 509 eyes). There was no significant difference in IOP between the two groups (WMD: 1.44, 95 % CI: −0.76, 3.65; *p* = 0.20) and significant heterogeneity was observed (I^2^ = 74 %, *P* = 0.02). Adult subgroup analysis included the same studies as the RCT group in long-term follow-up.

### Use of glaucoma medications

**Fig. 6:**
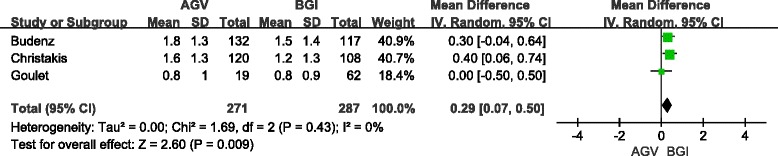
Forest plot of meta-analysis: medications in short-term follow-up

**Fig. 7:**
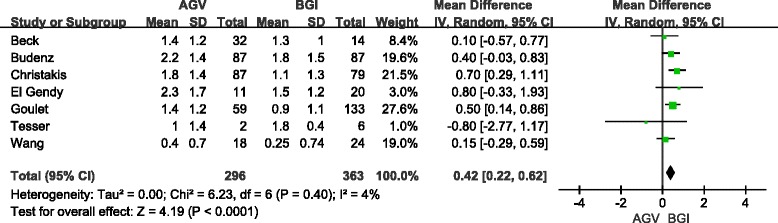
Forest plot of meta-analysis: medications in long-term follow-up

The mean number of glaucoma medications was reported by three studies (558 eyes) for short-term follow-up and seven studies (659 eyes) for long-term follow-up. Pooled differences showed that BGI implantation lowered the number of medications by a significant value of 0.29 (95 % CI: 0.07, 0.50; *P* = 0.009) in short-term follow-up (Fig. 6) and 0.42 (95 % CI: 0.22, 0.62; *P* <0.05) in long-term follow-up (Fig. 7). Sensitivity analysis and RCT subgroup analysis showed a significant difference in the mean number of glaucoma medications between the BGI and AGV groups in long-and short-term follow-up (Table 6). The random-effect model was used for pooling. One retrospective study (81 eyes) reported that medication use was not significantly different between the BGI and AGV groups. For the long-term follow-up, the pooled results of the non-RCT subgroups were consistent with the total group. No significant difference in use of glaucoma medication between the BGI and AGV groups was observed in the children subgroup (3 studies, 85 eyes). The WMD was 0.20 (95 % CI: −0.40, 0.80, *P* = 0.51). Adult subgroup analysis included the same studies as the RCT subgroup. The heterogeneity test showed a lack of significant heterogeneity for the total, subgroup, and sensitivity analyses.

**Table 6:** Using of medication in comparing AGV with BGI

Studies of subgroups	No. of studies	No. of eyes	WMD (95 % CI)	Heterogeneity		Test for over effect (*P*)
				I^2^ (%)	*P*	
Short-term follow-ups						
Total	3	558	0.29 (0.07–0.50)	0	0.43	0.009
RCT	2	477	0.35 (0.11–0.59)	0	0.68	0.004
Non-RCT	1	81	0.00 (−0.50–0.50)	/	/	1
Children	0	/	/	/	/	/
Adults	Same as RCT group					
Long-term follow-ups						
Total	7	659	0.42 (0.22–0.62)	4	0.40	0.000
Sensitivity analysis	6	651	0.43 (0.24–0.62)	0	0.45	0.000
RCT	2	340	0.56 (0.26–0.85)	0	0.32	0.000
Non-RCT	5	319	0.33 (0.08–0.57)	0	0.42	0.01
Children	3	85	0.20 (−0.40–0.80)	7	0.34	0.51
Adults	Same as RCT group					

### Postoperative complications

A total of 971 eyes (443 in the AGV group and 528 in the BGI group) were included in analysis of complications. Because Budenz et al. reported early (≤3 months) complications [21] and late (>3 months) complications [22], the latter category was used in the pooled calculations. The definition of severe complications was the same as that in the original studies, including severe complications and devastating complications. If the studies did not report numbers of severe or devastating complications, we included the following complications for pooling: suprachoroidal hemorrhage, severe choroidal effusion (requiring correctional surgery), retinal detachment, endophthalmitis, and vitreous hemorrhage. A total of 158 eyes in the AGV group and 199 eyes in the BGI group experienced complications. Eyes in the AGV group experienced a significantly lower overall occurrence of complications than those in the BGI group (OR, 0.67; 95 % CI: 0.50, 0.90; *P* = 0.007) and no heterogeneity was identified (I^2^ = 0 %, *P* = 0.88). The occurrence of severe complications in the AGV group was also lower than that in the BGI group (OR: 0.57; 95 % CI: 0.36, 0.91, *P* = 0.02). The AGV group was characterized by a lower incidence of hypotony, but this difference was not statistically significant (6 studies, 724 eyes; OR: 0.54; 95 % CI: 0.26, 1.11; *P* = 0.1). There were no significant differences in hyphema, choroidal effusion, and tube complications (including tube obstruction, malposition, and erosion) between the two groups. The results of sensitivity analysis were consistent with the total groups (included all eligible studies). The incidence of complications in both groups is listed in Table 7.

**Table 7:** Risk of complications in comparing AGV with BGI

Complications	No. of studies	Crude data (n/N)		OR (95 % CI)	Heterogeneity		Test for over effect (*P*)
		AGV	BGI		I^2^ (%)	*P*	
Total (eyes)	8	158/443	199/528	0.67 (0.50, 0.90)	0	0.88	0.007
Severe complication (cases)	8	34/443	58/528	0.57 (0.36, 0.91)	0	0.83	0.02
Hypotony (cases)	6	18/316	34/408	0.54 (0.26, 1.11)	5	0.38	0.1
Tube complication (cases)	4	28/317	40/303	0.68 (0.25, 1.87)	56	0.08	0.46
Hyphema (cases)	4	23/317	33/303	0.64 (0.36, 1.13)	0	0.58	0.12
Choroidal effusion (cases)	5	40/336	38/357	1.10 (0.72, 1.69)	0	0.62	0.66

Begg’s test and funnel plots were used to assess publication bias in pooled effect sizes that calculated using five or more studies. Publication bias assessment showed no significant bias in success rates, IOP, and glaucoma medications in long-term follow-up, overall and severe complications, hypotony, and choroidal effusion (all *P* ≥ 0.05).

We used GRADEpro 3.6 software to assess the quality of evidence for each outcome in the total groups (Table 8). Because data from RCTs and non-RCTs were included in the analysis, we used the standards of an observational study to assess overall outcomes. The pooled IOP and risk of tube complications were identified significant heterogeneity; therefore we graded it as “inconsistency”. We downgraded outcomes of tube complications, IOP in short-and long-term as “very low” quality. The rest of the outcomes were graded “low” quality.

**Table 8:** Summary of AGV compared to BGI for glaucoma

Outcomes	No of participants (studies) follow up	Quality of the evidence (GRADE)	Relative effect (95 % CI)	Anticipated absolute effects	
				Risk with BGI	Risk difference with AGV (95 % CI)
IOP (short-term)	685 (6 studies) 12 months	⊕⊝⊝⊝ VERY LOW^1^ due to inconsistency			The mean iop (short-term) in the intervention groups was 2.12 higher (0.72 to 3.52 higher)
IOP (long-term)	659 (7 studies) 20 to 60 months	⊕⊝⊝⊝ VERY LOW^2^ due to inconsistency			The mean iop (long-term) in the intervention groups was 1.85 higher (0.43 to 3.28 higher)
Success rate (short-term)	714 (5 studies) 12 months	⊕ ⊕ ⊝⊝ LOW	OR 0.97 (0.56 to 1.66)	797 per 1000	5 fewer per 1000 (from 110 fewer to 70 more)
Success rate (long-term)	835 (7 studies) 20 to 60 months	⊕ ⊕ ⊝⊝ LOW	OR 0.73 (0.54 to 0.99)	684 per 1000	72 fewer per 1000 (from 2 fewer to 145 fewer)
Medication (short-term)	558 (3 studies) 12 months	⊕ ⊕ ⊝⊝ LOW			The mean medication (short-term) in the intervention groups was 0.29 higher (0.07 to 0.5 higher)
Scale from: 0 to 5.					
Medication (long-term)	659 (7 studies) 20 to 60 months	⊕ ⊕ ⊝⊝ LOW			The mean medication (long-term) in the intervention groups was 0.42 higher (0.22 to 0.62 higher)
Scale from: 0 to 5.					
Total complications	971 (8 studies) 1 to 3 years	⊕ ⊕ ⊝⊝ LOW	OR 0.67 (0.5 to 0.9)	377 per 1000	89 fewer per 1000 (from 24 fewer to 145 fewer)
Servere complications	971 (8 studies) 1 to 3 years	⊕ ⊕ ⊝⊝ LOW	OR 0.57 (0.36 to 0.91)	110 per 1000	44 fewer per 1000 (from 9 fewer to 67 fewer)
Reoperation for glaucoma	632 (3 studies) 3 to 5 years	⊕ ⊕ ⊝⊝ LOW	OR 2.7 (1.54 to 4.74)	60 per 1000	87 more per 1000 (from 29 more to 172 more)
Hypotony	724 (6 studies) 1 to 3 years	⊕ ⊕ ⊝⊝ LOW	OR 0.54 (0.26 to 1.11)	83 per 1000	37 fewer per 1000 (from 60 fewer to 8 more)
Tube complications	620 (4 studies) 1 to 3 years	⊕⊝⊝⊝ VERY LOW^3^ due to inconsistency	OR 0.68 (0.25 to 1.87)	132 per 1000	38 fewer per 1000 (from 95 fewer to 89 more)
Hyphema	620 (4 studies) 1 to 3 years	⊕ ⊕ ⊝⊝ LOW	OR 0.64 (0.36 to 1.13)	109 per 1000	36 fewer per 1000 (from 67 fewer to 12 more)
Choroidal effusion	693 (5 studies) 13 to 36 months	⊕ ⊕ ⊝⊝ LOW	OR 1.1 (0.72 to 1.69)	106 per 1000	11 more per 1000 (from 30 fewer to 73 more)

*CI*: Confidence interval; *RR*: Risk ratio; *OR*: Odds ratio

GRADE Working Group grades of evidence

High quality: Further research is very unlikely to change our confidence in the estimate of effect

Moderate quality: Further research is likely to have an important impact on our confidence in the estimate of effect and may change the estimate

Low quality: Further research is very likely to have an important impact on our confidence in the estimate of effect and is likely to change the estimate

Very low quality: We are very uncertain about the estimate

^1^Significant heterogeneity was observed (*P* = 0.08)

^2^Significant heterogeneity was observed (*P* = 0.1)

^3^Significant heterogeneity identified (*p* = 0.08)

## Discussion

A total of 10 studies were included in this meta-analysis. Two of these studies were RCTs and eight were non-RCTs. The pooled results showed no statistically significant difference in success rates between the AGV and BGI groups for short-term follow-up. The success rates for the AGV group were lower than for the BGI group for long-term follow-up, but sensitivity and subgroup analyses showed a lack of stability. Nonetheless, the BGI group had better efficacy in controlling IOP than the AGV group. The pooled results from the RCT subgroup support the point that better efficacy in the BGI group, but the non-RCT subgroup showed negative results with significant heterogeneity. The BGI group required fewer glaucoma medications than the AGV group. More reoperations for glaucoma were required in the AGV group than in the BGI group. With regard to safety, the AGV was associated with a significantly lower overall frequency of adverse events and incidence of severe complications than the BGI. In subgroup analysis based on age, all of the studies that were included in children subgroup analyses were retrospective studies and sample sizes were small. More well-designed studies with a larger sample size needed to be performed in children. Publication bias and heterogeneity testing indicated that the pooled results were valid.

Although both implantations shared a similar success rate, the BGI resulted in a lower level of postoperative IOP and use of glaucoma medications than the AGV. The major success criteria, upper limit of IOP, ranged from 21 to 24 mmHg. However, the Advanced Glaucoma Intervention Study showed that an IOP target of greater than 18 mmHg may be insufficient to prevent progression of visual field defects [23]. Therefore, when setting a strict IOP target, the BGI may be more advantageous than the AGV. A larger surface area of the end plate for the Baerveldt implant (350 mm^2^ or 250 mm^2^) compared with the Ahmed valve (184 mm^2^) would theoretically help aqueous humor reabsorption into the circulation. Previous studies compared the efficacy of IOP control in several GDIs with different surface areas. They showed that the double-plate Molteno implant (surface area = 268 mm^2^) was superior to the single-plate implant [24]. The 350-mm^2^ Baerveldt implant was more successful than the 500-mm^2^ implant for overall IOP control [25]. These studies suggested that IOP control may be nonlinear relative to the surface area of the end plate. Although the AGV is equipped with a valve to reduce the occurrence of postoperative complications, the resistance to aqueous humor outflow eventually becomes counterproductive [26].

The models of the Ahmed valve and Baerveldt implant in our study were not consistent. Old polypropylene models (S2 and S3) and new silicone models (FP7) of the Ahmed valve were tested. Whether differences in biomaterial and end plate rigidity added an additional contribution to long-term IOP results was still uncertain, but the silicone model was associated with a lower incidence of complications [27–30]. Our study included the 350-mm^2^ model and the 250-mm^2^ model of BGIs, and both were made of silicone. Previous studies showed that these two models shared similar success rates and occurrence of complications [15, 31]. Despite the controversial effects of characteristics of the implant, potential heterogeneity from inconsistencies in the models could weaken the pooled results.

Begg’s test and funnel plots were used to assess publication bias. We found no significant bias. However, the results of the funnel plots may not be statistically meaningful because of the lack of power for the small sample size.

To minimize heterogeneity due to the inconsistencies of follow-up times, we pooled data for two time periods. Implantations concurrent with lens-related surgeries were enrolled in this meta-analysis. Phacoemulsification and extracapsular cataract removal can reduce IOP [32, 33], especially in patients with a shallow anterior chamber. However, the effects of these procedures combined with glaucoma implantation devices were uncertain. In addition, extra surgical procedures could lead to a higher risk of adverse events. Despite this heterogeneity, we included these two articles because they provided important clinical information. Furthermore, sensitivity analysis was performed to examine the heterogeneity.

There are some limitations to our study. First, only two RCTs were included in the studies. Most studies were retrospective comparative studies that had a potential selection bias. A small sample size and incomplete baseline data also weakened the validity of the tests. Second, surgical success and complication criteria were not standardized among the included studies. Therefore, standardized assessment criteria should be established in further studies. Third, our current statistical methodology assumed that the input samples were approximately symmetric and approximately followed a Gaussian distribution. However, the values of glaucoma medications are non-negative integers, mostly in the range of 1 to 4, which are more likely to have a skewed distribution. Skewed distributions tend to have larger SD than mean. This over-generalized assumption may result in biased conclusion. Fourth, we did not analyze visual outcomes as a result of inconsistent statistical methods used in the visual results. Furthermore, we did not perform subgroup analyses for types of glaucoma and race.

When choosing a device, other factors should also be considered, for example, the experience of the surgeon, compliance during follow-up, and the goals for therapy. Moreover, additional RCTs with a longer duration and a larger sample size are required to better determine the efficacy and safety of the AGV and BGI for the treatment of glaucoma.

## Conclusions

This study showed no significant difference in the success rate between the BGI and AGV groups. The BGI performed better in the control of IOP and required fewer medications than the AGV. The AGV performed better than the BGI regarding safety.

## Additional files

Additional file 1:
**Search strategy.doc, search strategy for meta-analysis.** (DOCX 16 kb) Additional file 2:
**PRISMA 2009 Checklist.doc, PRISMA Checklist for meta-analysis.** (DOC 62 kb)

## Abbreviations

AGV: Ahmed glaucoma valve
BGI: Baerveldt glaucoma implant
CI: Confidence interval
GDI: Glaucoma drainage implant
GRADE: Grading of recommendations assessment, development and evaluation
IOL: Intraocular lens
IOP: Intraocular pressure
non-RCT: Non-randomized controlled clinical trial
OR: Odds ratio
RCT: Randomized controlled clinical trial
WMD: Weighted mean difference
