# Supplementary material for: Comparison of the Ahmed glaucoma valve with the Baerveldt glaucoma implant: a meta-analysis
Source: BMC Ophthalmol. 2015 Oct 13;15:132. doi: 10.1186/s12886-015-0115-y (PMC4605098; doi:10.1186/s12886-015-0115-y)
Supplement: Additional file 1: — Search strategy.doc, search strategy for meta-analysis. (DOCX 16 kb) [file 12886_2015_115_MOESM1_ESM.docx]

Search strategy for each Database：

Pubmed

1. "Glaucoma"[Mesh]
2. "Ocular Hypertension"[Mesh]
3. "Intraocular Pressure"[Mesh]
4. Glaucoma*[tiab]
5. Or/1-4
6. Ahmed[tiab]
7. Baerveldt[tiab]
8. 5 and 6 and 7

EMBASE

1. ‘glaucoma’/exp
2. ‘intraocular hypertension’/exp
3. ‘intraocular pressure’/exp
4. ((increase* or elevat* or high*) near/3 ocular near/3 pressure):ab,ti
5. Glaucoma*:ti,ab
6. Or/1-5
7. Ahmed:ti,ab
8. Baerveldt:ti,ab
9. 6 and 7 and 8

Cochrane

1. MeSH descriptor Glaucoma
2. MeSH descriptor Ocular Hypertension
3. MeSH descriptor Intraocular Pressure
4. (pressure near ocular) and (increas* or elevat* or high*)
5. glaucoma*:ti,ab
6. or/1-5
7. Ahmed:ti,ab
8. Baerveldt:ti,ab
9. 6 and 7 and 8

Time：2015-02-27

PubMed: 49

EMBASE: 52

Cochrane: 10

Total：111

After duplicates removed：54
